# Amelioration of murine sickle cell disease by nonablative conditioning and γ-globin gene-corrected bone marrow cells

**DOI:** 10.1038/mtm.2015.45

**Published:** 2015-12-02

**Authors:** Tamara I Pestina, Phillip W Hargrove, Huifen Zhao, Paul E Mead, Matthew P Smeltzer, Mitchell J Weiss, Andrew Wilber, Derek A Persons

**Affiliations:** 1 Department of Hematology, St. Jude Children’s Research Hospital, Memphis, TN, USA; 2 Department of Pathology, St. Jude Children’s Research Hospital, Memphis, TN, USA; 3 Department of Epidemiology and Biostatistics, University of Memphis School of Public Health, Memphis, TN, USA; 4 Department of Medical Microbiology, Immunology and Cell Biology and Simmons Cancer Institute, Southern Illinois University School of Medicine, Springfield, IL, USA

## Abstract

Patients with severe sickle cell disease (SCD) are candidates for gene therapy using autologous hematopoietic stem cells (HSCs), but concomitant multi-organ disease may contraindicate pretransplant conditioning with full myeloablation. We tested whether nonmyeloablative conditioning, a regimen used successfully for allogeneic bone marrow transplantation of adult SCD patients, allows engraftment of γ-globin gene-corrected cells to a therapeutic level in the Berkeley mouse model of SCD. Animals transplanted according to this regimen averaged 35% engraftment of transduced hematopoietic stem cells with an average vector copy < 2.0. Fetal hemoglobin (HbF) levels ranged from 20 to 44% of total hemoglobin and approximately two-thirds of circulating red blood cells expressed HbF detected by immunofluorescence (F-cells). Gene therapy treatment of SCD mice ameliorated anemia, reduced hyperleukocytosis, improved renal function, and reduced iron accumulation in liver, spleen, and kidneys. Thus, modest levels of chimerism with donor cells expressing high levels of HbF from an insulated γ-globin lentiviral vector can improve the pathology of SCD in mice, thereby illustrating a potentially safe and effective strategy for gene therapy in humans.

## Highlights

- Nonmyeloablative conditioning allowed therapeutic engraftment of γ-globin gene-corrected cells in SCD mice.
- All transplanted SCD mice had > 20% HbF and reduced pathologies suggesting a safe and effective strategy for treating human SCD.

## Introduction

Sickle cell disease (SCD) is caused by a germ-line mutation that introduces a glutamic acid-to-valine substitution at the sixth coding amino acid of the β-globin protein. The resultant alteration in charge and hydrophobicity renders deoxygenated sickle hemoglobin (HbS; α_2_,β^S^_2_) susceptible to polymerization, causing red blood cells (RBCs) to become rigid and sickle-shaped. Consequently, sickle RBCs occlude small and medium blood vessels resulting in tissue hypoxia, pain crises, and organ damage.^1–3^ The symptoms of SCD develop during the first years of life coincident with the switch from fetal hemoglobin (HbF; α_2_,γ_2_) to adult hemoglobin (HbA; α_2_,β_2_) production in RBC precursors. SCD affects millions of people worldwide, causing substantial morbidity and mortality.^1–3^

The treatments for SCD are blood transfusions^2,3^ and hydroxyurea,^4^ which is believed to act, at least in part, by inducing HbF. Hydroxyurea is widely used for the treatment of SCD following clinical trials, which demonstrated its ability to reduce pain crisis, acute chest syndrome, and transfusion requirements for many patients.^5,6^ While this option can improve quality and duration of life,^7^ a significant number of patients do not benefit from hydroxyurea therapy due to suboptimal HbF responses and/or side effects.^8–10^ Allogeneic bone marrow (BM) transplantation from human leukocyte antigen (HLA)-matched donors can cure SCD.^11^ However, only about 20% of the patients have matched donors and mortality rates up to 10% can occur from infection and graft-versus-host disease.^11^ BM transplantation using alternative donor sources such as HLA-matched unrelated donors,^12^ HLA-mismatched family members,^13^ and unrelated umbilical cord blood units^14^ are under investigation, but these protocols are associated with a relatively high risk for serious complications for many SCD patients. These limitations of current curative therapies make gene replacement/correction in autologous hematopoietic stem cells (HSCs) a highly desired alternative.

Clinical evidence indicates that expression of γ-globin, which binds α-globin to form HbF, lessens the severity of SCD,^15–18^ partly because heteromeric (α_2_,β^S^γ) hemoglobin tetramers do not polymerize.^2,18^ Endogenous expression of HbF in SCD patients is variable and subject to genetic regulation by numerous loci including the β globin locus itself (*HBB*) and transcription factors regulating the γ-to-β switch (*BCL11A*, *MYB*, and *KLF1*).^19^ In general, SCD patients with endogenous HbF ≥ 20% experience reduced symptoms.^10^ In extreme cases, termed hereditary persistence of fetal hemoglobin (HPFH), γ-globin is not completely silenced and HbF is produced in most or all adult RBCs at levels of > 30%.^15,20,21^ Individuals with compound heterozygosity for HbS and HPFH alleles are usually asymptomatic.

The benefits of HbF suggest that therapeutic improvements in SCD may be achieved through ectopic expression of γ-globin in autologous HSCs via gene therapy approaches. Lentiviral vectors are currently the most effective mode of gene delivery to HSCs for achieving high-level, erythroid-restricted expression. The most effective designs contain the human β-globin locus control region (LCR) and promoter, a γ- or β-globin protein-coding gene and *ΗΒΒ* 3’ enhancer sequences, all in reverse orientation.^22^ Additional modifications include the insertion of chromatin insulator elements in the 3’ long terminal repeat (LTR) to confer barrier and enhancer blocking activities.^23^ We and others have used lentiviral vectors encoding human γ-globin or β-globin derivatives to improve^24^ or correct^25–27^ mouse models of SCD. In these studies, therapeutic benefit was achieved when animals received a lethal dose of radiation prior to transplant with genetically modified HSCs. However, many SCD patients have preexisting multiorgan disease, which may increase the risk of full myeloablative transplant regimens.^28^ One study examined the beneficial effects of autologous HSC gene therapy following sublethal conditioning; however, survival and benefit was dependent upon supportive RBC transfusions and some recipients did not achieve therapeutic expression of the γ-globin transgene.^27^ Therefore, additional efforts are needed to refine subablative conditioning approaches for SCD gene therapy.

Recently, allogeneic transplant protocols combining nonmyeloablative conditioning with rapamycin (RAPA) immunosuppression have been successful in adult SCD patients using HLA-matched donors.^29,30^ In this study, we tested whether these conditioning techniques could be used successfully to support engraftment of HSCs transduced with a SCD therapeutic vector. We have developed an insulated, self-inactivating (SIN) lentiviral vector encoding for erythroid-specific expression of γ-globin genomic sequences (termed V5m3-400). We demonstrated that transduction of SCD BM CD34^+^ cells with this vector reduced deoxygenation-induced sickling of *in vitro*-derived RBC progeny.^31^ To further support use of the V5m3-400 vector in SCD clinical trials, we tested whether SCD mice receiving nonablative irradiation would engraft with γ-globin gene-corrected HSCs to a level sufficient for a therapeutic effect. Transplanted animals averaged 35% HSC chimerism and had HbF levels ≥ 20% in circulating RBCs, which improved anemia and reduced SCD-related pathologies. Our results confirm and extend prior work showing that lentiviral gene therapy for SCD with nonablative conditioning to achieve modest chimerism levels of transduced HSCs is feasible and effective in mice, supporting similar strategies for treating patients.

## Results

### High HbF following γ-globin lentiviral vector gene therapy and nonmyeloablative transplantation in SCD mice

**Figure 1:**
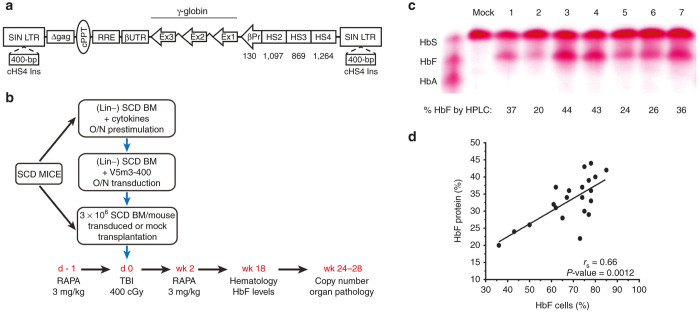
Transplantation of sickle cell disease (SCD) mice with V5m3-400 γ-globin vector-transduced sickle hematopoietic stem cells (HSCs) after subablative conditioning promotes high level HbF expression. (**a**) Schematic of the integrated provirus genome for the insulated, self-inactivating (SIN) γ-globin vector (V5m3-400). cPPT, central polypurine tract; RRE, Rev-responsive element; UTR, human β-globin 3’ untranslated sequences; Ex, ^A^γ-globin coding exons; βp, human β-globin promoter; HS, DNase1 hypersensitive sites; cHS4; 400-bp core element of chicken HS4 insulator; LTR, long terminal repeat. (**b**) Experimental schema for transduction and transplantation of sickle bone marrow (BM) HSC into preconditioned SCD mice. Lineage-negative BM cells (Lin-) from SCD mice were prestimulated and transduced overnight (O/N) with V5m3-400 lentivirus particles to achieve a multiplicity of infection (m.o.i.) of 40. Recipient SCD mice were given rapamycin (RAPA) intraperitoneally (i.p.) 1 day before (d -1) irradiation. On day 0, mice received 400 cGy total body irradiation and were transplanted with 3 × 10^6^ HSC/progenitor cells transduced under mock conditions or with the V5m3-400 vector. RAPA treatment was reinitiated the day after transplant and continued once daily for 2 weeks. HbF production, vector copy number and organ pathology determined at the indicated times. (**c**) Cellulose acetate hemoglobin gel demonstrating Hb species in lysates of whole blood collected from mice 18 weeks post-transplantation with sickle HSC transduced using mock conditions (lane 1) or with the V5m3-400 vector (lanes 2–7). A standard consisting of equal amounts of HbS, HbF and HbA is shown (left) and the percentage of HbF as quantified by high-performance liquid chromatography reported below each lane. (**d**) Percentages of RBC HbF versus circulating F-cells measured at 18 weeks post-transplantation. Regression lines (*r*_s_) and *P* values were calculated by Spearman’s rank order analysis. Each symbol represents an individual mouse.

Previously, we demonstrated high-level production of HbF in lethally irradiated C57Bl/6 mice transplanted with lineage-depleted (Lin-) SCD BM cells transduced with a γ-globin lentiviral vector, termed V5m3.^26^ The V5m3 vector encodes 3.1-kb of sequences from the β-globin LCR and a 130-bp β-globin promoter driving transcription of γ-globin genomic sequences in which the endogenous 3’-untranslated region is replaced with its β-globin counterpart. We also introduced 400-bp of core sequences from the chicken β-globin HS4 insulator element into the deleted U3 portion of the 3’-LTR (Figure 1a). This vector, termed V5m3-400, directed slightly higher HbF production in erythroblasts derived from transduced CD34^+^ cells of healthy donors compared to V5m3.^32^

Three independent nonmyeloablative transplant experiments were performed in the Berkeley (BERK) mouse model of SCD (Figure 1b). Lineage-depleted BM cells from BERK SCD mice (CD45.1 or CD45.2) were enriched by immunomagnetic separation, prestimulated overnight, and transduced under mock conditions or with VSV-G psuedotyped V5m3-400 vector particles to achieve a multiplicity of infection of ~40. Mock conditions were chosen over a control vector (*e.g.*, GFP reporter) to assess optimal engraftment potential of long-term hematopoietic repopulating cells following nonablative conditioning. To determine HSC gene transfer efficiency of the V5m3-400 vector, we injected a fraction of the transduced cells into irradiated mice, harvested spleen colonies (CFU-S) and screened them for vector genomes by Southern blotting. In three experiments, 41/48 (85%) of CFU-S colonies contained vector DNA (not shown). For BM transplantation studies, mice from 1 to 4 months old received 400 cGy total body irradiation and were infused with 3 × 10^6^ mock-transduced or V5m3-400-transduced cells. The donor (CD45.1 or CD45.2) and recipient (CD45.1/CD45.2 mixed) BERK SCD mice were on mixed genetic backgrounds and therefore not syngeneic (see Materials and Methods). In pilot studies, we determined that engraftment of lentiviral transduced donor cells was dependent upon immunosuppression of recipient mice with RAPA (Supplementary Figure S1). Therefore, recipient mice were immunosuppressed with RAPA (3 mg/kg i.p.) on days −1 and +1–14 to promote T-cell tolerance and mixed chimerism.^33^ Animals were analyzed at 4–5 months post-transplant for complete blood counts and HbF expression in peripheral blood RBC and at 6–7 months post-transplant to determine HSC gene transfer efficiencies, renal function, as well as pathology and iron accumulation in liver, spleen, and kidneys.

Table 1 shows the salient features of gene therapy-treated mice. Engraftment of donor cells was 60 ± 16% (mock) versus 35 ± 14% (V5m3-400) based on flow cytometry analysis of peripheral blood lymphocytes for the expression of CD45.1 and/or CD45.2. Immunostaining of RBCs with an anti-HbF antibody demonstrated that 36–85% of cells expressed the vector-encoded γ-globin transgene. Average vector copy number (VCN) was 0.6 ± 0.3 determined by Southern blot analysis of BM DNA from transplanted animals. HbF constituted 20–44% of the total hemoglobin in RBC lysates, as analyzed by cellulose acetate gel electrophoresis and high-performance liquid chromatography (Figure 1c). The proportion of F-cells correlated with HbF production with each F-cell expressing ~50% HbF (Figure 1d); however, total Hb lacked correlation with VCN (Supplementary Figure S2). Levels of F-cells and HbF in peripheral blood remained stable throughout the post-transplant period (up to 7 months). Blood samples for several mice were stained with thiazole orange to identify reticulocytes using flow cytometry. Consistent with previous γ-globin gene replacement studies using SCD mouse models,^26,27^ the proportion of reticulocytes decreased from 35% (range 25–48%) in mock mice to 5% (range 4–9%) in V5m3-400-treated mice (*P* = 0.0007). Blood samples were also incubated with antibodies to transferrin receptor (CD71), then permeabilized and stained for intracellular HbF to determine reticulocyte chimerism by flow cytometry. As expected, CD71^+^ cells from mock mice lacked HbF expression, while 25% of CD71^+^ cells of V5m3-400-treated mice coexpressed HbF. The proportion of reticulocytes expressing HbF correlated with F-cells (Supplementary Figure S3a). However, within each animal, the %F cells were twofold greater than the %F reticulocytes, indicating that HbF expression increases the lifespan of circulating RBCs. In agreement, *in vivo* biotin-labeling studies demonstrated that the RBC half-life was significantly improved in V5m3-400-treated mice compared to mock-treated mice (Supplementary Figure S3b). Flow cytometry analysis of peripheral blood samples collected 6 days after injection showed mean percentages of biotin-labeled RBCs were threefold higher for gene therapy-treated mice (24 ± 1.2% biotin^+^; mean ± SEM) versus mock controls (7.7 ± 2.8%; *P* = 0.0004 by *t*-test).

### High-level HbF expression ameliorates the anemia, associated leukocytosis, and secondary organ damage of SCD

**Figure 2:**
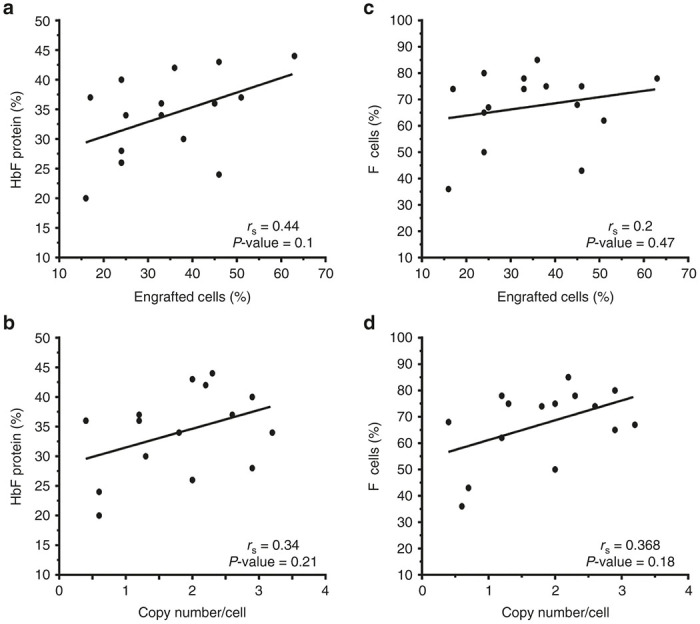
Relationship between percentage engraftment and vector copy number to levels of HbF and F-cells in peripheral blood of gene therapy treated sickle cell disease mice. Percentages of RBC HbF (**a**,**b**) or circulating F-cells (**c**,**d**) versus % donor engraftment (**a**,**c**) or vector copy number per cell (**b**,**d**) measured in circulating mononuclear cells at 18 weeks post-transplantation. Regression lines (*r*_s_) and *P* values were calculated by Spearman’s rank order analysis. Each symbol represents an individual mouse.

**Figure 3:**
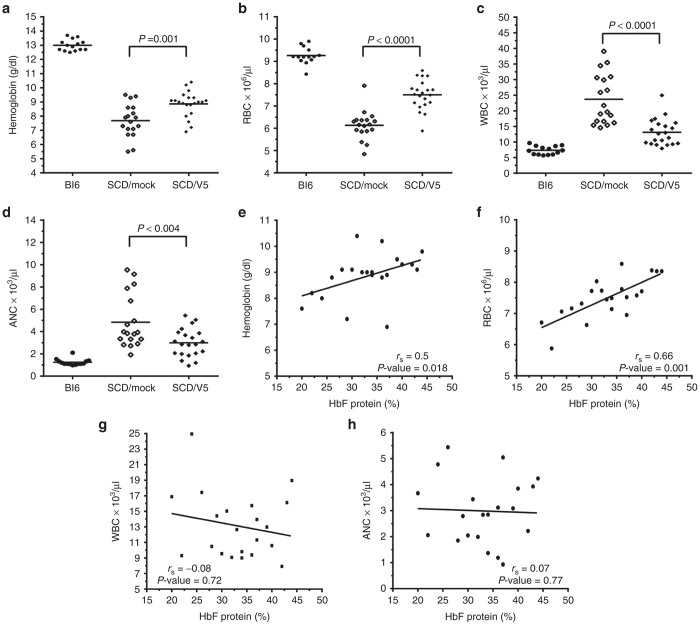
V5m3-400 gene therapy ameliorates anemia and hyperleukocytosis in sickle cell disease (SCD) mice. Panels (**a**–**d**) show blood hemoglobin (Hb) levels (**a**), red blood cell (RBC) counts (**b**), white blood cell (WBC) counts (**c**), and absolute neutrophil counts (ANC) (**d**), for untreated C57Bl6 (Bl6; *n* = 14) mice or SCD mice transplanted with SCD HSC/progenitors that were mock-transduced (SCD/mock; *n* = 18) or transduced with the V5m3-400 vector (SCD/V5; *n* = 21). Panels (**e**–**h**): parameters indicated on the Y-axis and corresponding to the graphs (panels **a**–**d**) are plotted against % HbF. Each symbol represents an individual mouse. Mean values for each group in (**a**–**d**) are represented by the horizontal line and *P* values were calculated by two-tailed Student’s *t*-test. In panels (**e**–**h**) regression lines (*r*_s_) and *P* values were calculated by Spearman’s rank order analysis.

In patients with SCD, HbF levels exceeding 20% are believed to produce substantial clinical benefits.^20^ All gene therapy-treated mice met or exceeded this level. HbF protein (Figure 2a,b) correlated better with the proportion of engrafted cells and vector copies compared to F-cells (Figure 2c,d), with a trend toward significance. Table 2 shows hematological indices in gene therapy-treated versus mock-transduced SCD mice and wild-type C57Bl/6 mice. High levels of HbF led to significant increases in the Hb and RBC counts, compared to mice that received mock-transduced cells (Figure 3a,b and Table 2). Elevated white blood cell (WBC) counts in humans with SCD and BERK mice reflect inflammation that likely contributes to organ damage. Gene therapy-treated mice showed significant reduction in WBC counts compared to mock-transduced controls (Figure 3c and Table 2). Similar improvement was observed using the independent measure of absolute neutrophil counts (ANC) (Figure 3d and Table 2). Correlation was found between HbF and blood Hb concentration or RBC count (Figure 3e,f). However, a similar relationship was not identified between HbF and WBC or ANC (Figure 3g,h).

**Figure 4:**
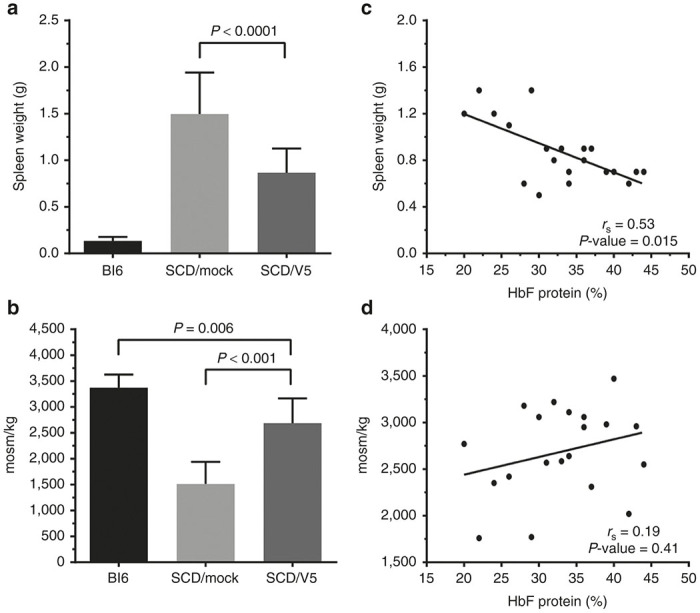
Protection against secondary organ damage in gene therapy treated mice. Spleen weight in grams (g) (**a**,**c**) and urine concentrating ability (**b**,**d**) were measured for untreated C57Bl/6 (Bl6; *n* = 5) mice in control or gene therapy treated sickle cell disease (SCD) mice, as described in Figures 1 and 2 (SCD/mock, *n* = 19; SCD/V5, *n* = 21). The right panels show spleen weight (**c**) or urine concentrating ability (**d**) plotted against % HbF in RBCs. Data were obtained 18 weeks post-transplantation. The left panels show the mean ± SD for each group with *P* values calculated by two-tailed Student’s *t*-test. In the right panels, each symbol represents an individual mouse with regression lines (*r*_s_) and *P* values calculated by Spearman’s rank order analysis.

Splenic enlargement occurs in young SCD patients and in BERK mice, reflecting increased erythropoietic drive resulting from the reduced half-life of sickled RBCs.^34^ Mice transplanted with V5m3-400 gene-corrected HSCs demonstrated a significant reduction in spleen weight compared to mock controls (Figure 4a and Table 2). In older SCD patients, nephropathy is a problem, with ~5% of patients developing end stage renal disease.^35^ One of the earliest manifestations of kidney damage in SCD patients is defective urine concentrating ability, also observed in BERK mice. Gene therapy-treated animals demonstrated significant improvement in urine concentrating ability compared to the mock group (Figure 4b and Table 2). Correlation was found between HbF and spleen weight (Figure 4c); however, a similar relationship was not identified between HbF and urine concentrating ability (Figure 4d).

**Figure 5:**
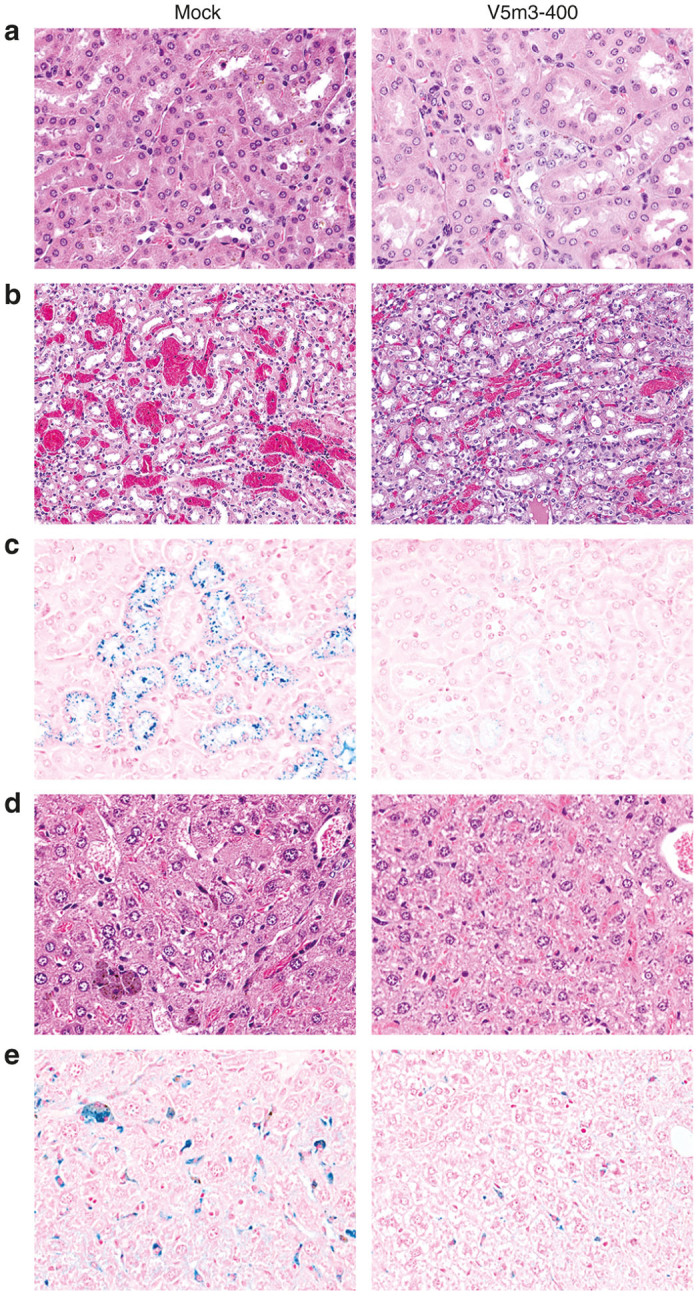
Reduction of excessive kidney pathology and iron accumulation in kidneys, liver, and spleen of sickle cell disease (SCD) mice transplanted with V5m3-400 vector-transduced hematopoietic stem cells (HSCs). SCD mice transplanted with SCD stem cells that were mock-transduced (mock) or transduced with the V5m3-400 vector (V5m3-400) were sacrificed 24–28 weeks post-transplantation. Tissue sections were stained to evaluate organ pathology and iron accumulation and photographed at ×20 (panel b) or ×40 magnification (panels **a**,**c**–**e**). (**a**) Representative hematoxylin and eosin (H&E)-stained sections of renal tubular epithelium showing hemosiderin/iron accumulation in proximal tubules. (**b**) Examples of H&E stained kidney tissues illustrating the greater degree of congestion/dilation of medullary capillaries for mock-transduced mice. (**c**) Examples of proximal renal tubules stained for iron content with Prussian blue showing reduced iron deposition in kidneys of mice transplanted with V5m3-400 vector-transduced HSC. (**d**) Clusters of hypertrophic macrophages and Kupffer cells distended with hemosiderin are evident in H&E stained sections of liver from mock-transduced mice. (**e**) Liver sections stained with Prussian blue shows reduced iron deposition in mice transplanted with V5m3-400 vector-transduced HSCs.

We collected spleen, kidney, and liver tissues from a subset of animals for mock and V5m3-400 treatment groups to evaluate further the effects of gene therapy on SCD-related organ pathology. Animals examined had engrafted SCD donor cells of 56 ± 21% (mock, *n* = 7 of 18 total) versus 36 ± 11% (V5m3-400, *n* = 12 of 21 total). The HbF derived from the vector-encoded γ-globin transgene in RBC lysates ranged from 24 to 44% of the total hemoglobin, averaging 34%. Total Hb levels for V5m3-400-treated mice were 9.1 ± 0.6 g/dl compared to 7.5 ± 1.7 g/dl for the mock group, and RBC counts were similarly increased for the V5m3-400 group (7.7 ± 0.5 × 10^6^/µl) versus mock controls (6.1 ± 0.6 × 10^6^/µl). Thus, sampled animals were representative of collective treatment groups (see Table 2). Mock-treated animals exhibited numerous SCD-related pathologies including splenic erythroid hyperplasia and dilated sinusoids containing pools of sickled RBCs (not shown). The lesions in the kidney of mock-treated mice included hemosiderin/iron accumulation in proximal tubules (Figure 5a) and congestion/dilation of medullary capillaries (Figure 5b). There was iron deposition in the kidney glomeruli (Figure 5c) reflecting chronic hemolysis. The livers of mock-treated animals showed clusters of hypertrophic macrophages and Kupffer cells stained with hemosiderin (Figure 5d) and increased intracellular iron content by Prussian blue (Figure 5e). These abnormalities were still present in the gene therapy-treated animals, but the severity was markedly decreased.

Before the age of 1 year, SCD patients are relatively protected from anemia and organ pathology by increased HbF levels.^36,37^ BERK SCD mice are anemic at an early age, as indicated by reduced total Hb and RBC counts compared to wild-type Bl6 mice and heterozygous SCD (SCD Hz) controls (Supplementary Figure S4a,b). Hb levels declined as animals aged, but RBC counts, while significantly lower than control groups, were essentially unchanged with age. WBC and ANC counts, indicators of inflammation, were significantly increased in older animals (Supplementary Figure S4c,d). Because younger mice demonstrated reduced disease-related inflammation, we tested for association between age of recipient at the time of BM transplantation and therapeutic efficacy (Table 3). Significant correlation was found between age and gene transfer (VCN/cell), WBC, ANC, and spleen size (Supplementary Figure S5a–d). In contrast, there was no correlation between recipient age and gene therapy-related improvement in HbF production, F-cell numbers, total Hb, RBCs, or renal concentrating ability (not shown).

## Discussion

Autologous stem cell gene therapy is an attractive option for treating SCD due to limited pharmacological options and restricted numbers of HLA-matched donors. Preclinical studies in SCD mouse models^24–27^ and human SCD erythroid cultures^31,38^ using lentiviral vectors capable of high-level, erythroid-specific expression of γ-globin or β-globin derivatives suggest that curative levels of antisickling Hb production are possible. In SCD mouse experiments, wild-type animals received a lethal dose of radiation prior to transplant with genetically modified SCD HSCs, resulting in complete engraftment of donor cells. While full myeloablative conditioning has been successful in pediatric SCD patients,^39–41^ these protocols are relatively contraindicated for most adult patients due to higher risk of transplant-related morbidity and mortality associated with baseline disease-related organ dysfunction.^28^ Stable mixed chimerism has been achieved using nonmyeloablative conditioning protocols for animal transplant models that favor graft rejection.^42–44^ Recently, clinical protocols based on this regimen have been successful for BM transplantation of adult SCD patients with HLA-matched family donors.^29,30^ Here, we report that a nonmyeloablative conditioning regimen followed by transplantation of SCD HSCs and progenitors transduced with γ-globin expressing lentivirus results in mixed BM chimerism with amelioration of anemia and organ pathology. Although BM HSC chimerism was relatively low, HbF expression and % F-cells reached therapeutic levels because ectopic γ-globin expression conferred a survival advantage to circulating RBCs. Of note, our study utilized donor and recipient mice with different CD45 alleles to track levels of chimerism after BM transplantation. The complex genetic structure of the BERK SCD mice inhibited our ability to create syngeneic strains. Therefore, recipient mice were treated with rapamycin to inhibit graft rejection predicted to arise from minor histocompatibility differences with donor cells. For future SCD patients who receive similar therapies, immunosuppression should not be required since the BM transplantation will be autologous.

Our data confirm and extend an earlier study illustrating the beneficial effects of autologous HSC gene therapy in BERK SCD mice after sublethal irradiation to facilitate mixed chimerism.^27^ In that report, lineage negative, c-kit^+^, Sca-1^+^ (LSK) BM cells were transduced with lentivirus particles encoding an erythroid specific β-γ-globin hybrid transgene. In vector-treated mice, post-transplant survival depended on supportive RBC transfusions within the first 7 days after transplant. In agreement with our findings, SCD symptoms were relieved in mice with >10% HbF. However, this threshold was reached in only about 35% of treated mice. In the current study, animals did not require transfusions, probably because we used a lower radiation dose (400 versus 800 cGy) and because the donor cells were less highly purified (Lin- only) and therefore contained a higher proportion of intermediate-term repopulating cells to sustain erythropoiesis prior to long-term HSC engraftment. Potential reasons for improved HbF expression in our study include differences in vector design that impact viral titers and/or erythroid gene expression after integration. Both vectors utilized a similar SIN LV backbone with a γ-globin-like gene driven by sequences from the β-globin locus LCR, the β-globin promoter and 3’ enhancer. Variations between the promoter and/or LCR sequences used in these two vectors may be responsible for their observed differences in performance.

The V5m3-400 vector described here compares favorably when directly compared to other vectors designed for treating SCD.^31^ The uninsulated version of this vector (V5m3) raised the RBC Hb concentration by 4.1 g/dl/vector copy when lethally irradiated wild-type C57Bl/6 mice were fully engrafted with γ-globin gene-corrected SCD HSCs.^26^ This Hb rise was approximately twice that achieved in the same or similar SCD mouse models using different vectors.^24,25^ These include a SIN LV vector encoding a mutant form of β-globin (β^A-T87Q^) with anti-sickling activity that was subsequently tested in a phase 1/2 trial for severe β thalassemia in which one of three subjects became transfusion-independent.^45,46^ This clinical grade vector (termed LentiGlobin HPV569) included two copies of a 250-bp cHS4 insulator including a cryptic splice acceptor that led to activation of HMGA2 and clonal but stable hematopoiesis.^45^ A redesigned version of this vector with CMV promoter/enhancer replacing the 5’ HIV U3 portion of the LTR and without the insulator sequences, termed LentiGlobin BB305, exhibits improved titer and transduction efficiency^47^ and produced promising preliminary results in a clinical trial for β-thalassemia major.^48^ Of note, insulator elements in the V5m3-400 vector were found to be stably transmitted in target cells and in reconstituted cells in two treated subjects enrolled in an ongoing X-SCID clinical trial run at NIAID (B. Sorrentino, personal communication, 2015), and we have observed no evidence of clonal hematopoiesis in the treated mice (not shown). However, comparative studies are required to evaluate the ability of the 400-bp insulator to confer protection against integration position silencing of transgene expression in this model.

Overall, we found that modest donor chimerism with V5m3-400-transduced HSCs resulted in consistent HbF levels ≥ 20%, a threshold that likely produces substantial therapeutic effects in SCD.^21,49–51^ Moreover, approximately two-thirds of the gene therapy-treated mice exhibited HbF levels ≥ 30%, which was associated with additional improvements in some clinical parameters including Hb level and splenomegaly. In humans with homozygous SCD mutations, pancellular HbF with levels ≥ 30% are associated with complete absence of symptoms.^21,52^ Importantly, our results were achieved with a relative low VCN (0.4–3.2 copies per engrafted cell), which is compatible with a major goal for HSC-targeted gene therapy to obtain functional cell correction with the lowest VCN possible so as to avoid genotoxic risks.^53^ In this regard, we observed no evidence of myelodysplasia or leukemia in V5m3-treated animals in a prior study^26^ or in the current study over an observation period of 24–28 weeks post-transplantation.

Although an autologous gene therapy trial for SCD has not yet been reported in a peer reviewed report, several studies are recently initiated or planned for the near future.^54^ These efforts will be refined and improved over time with new advances in methods of identifying permissive HSC populations,^55^ gene delivery, genetic manipulation,^56^ and transplantation protocols. In this model, therapeutic levels of HbF were achieved by transplanting SCD mice with high numbers of cells (3 × 10^6^ Lin- cells) that had been effectively transduced (~85% of CFU-S vector positive). Still, donor chimerism was modest averaging 35% following nonablative conditioning. It could be argued that equivalent cell doses and transduction efficiencies would not be possible in the clinical situation. Importantly, these studies define the relationship between transduction efficiency and conditioning. As methods are developed to improve gene transfer efficiency in the setting of autologous gene therapy, the more sparing clinical protocols can be with conditioning. The current study describes a potent lentiviral vector that directs high-level erythroid γ-globin gene expression and along with the work of Perumbeti *et al*.,^27^ provides preclinical evidence that non-myeloablative conditioning with stable chimerism of gene-corrected cells may be a viable therapeutic strategy, particularly for many older SCD patients with organ damage who are at increased risk for toxicities associated with high doses of irradiation or myelotoxic drugs.

## Materials and Methods

### Lentiviral vector design and production

The details regarding the construction of the V5m3-400 γ-globin lentiviral vector used in these studies have been described.^32^ Lentiviral vector particles pseudotyped with vesicular stomatitis virus glycoprotein (VSV-G) were prepared and titrated as described;^57^ average titer was 3.9 × 10^8^ transducing units/ml.

### Sickle cell disease mice

The Berkeley (BERK) SCD mice have been engineered for production of HbS and previously used by our group for gene therapy studies.^26^ Homozygous SCD mice express three types of lymphocyte common antigen: CD45.1, CD45.2, and mixed CD45.1/CD45.2. In our studies, SCD mice expressing CD45.1 or CD45.2 alone were used as BM donors and mixed CD45.1/CD45.2 as recipients. Animal experiments were approved by the St. Jude Children’s Research Hospital Institutional Animal Care and Use Committee.

### Isolation, transduction, and transplantation of SCD bone marrow cells

The procedures for BM collection, lineage depletion, and prestimulation are as previously described.^26^ Prestimulated, lineage negative (Lin-) SCD donor cells (CD45.1 or CD45.2) were added to 12-well suspension plates coated with RetroNectin (Takara, Shiga, Japan) at 10–11 × 10^6^ cells/ml in StemSpan medium (StemCell Technologies, Vancouver, British Columbia, Canada) supplemented with 10 µg/ml heparin (Sigma-Aldrich, St. Louis, MO), 50 U/ml penicillin and streptomycin, 2 mmol/l glutamine and 10 ng/ml mouse stem cell factor, 50 ng/ml mouse thrombopoietin, 20 ng/ml mouse insulin-like growth factor 2 (all from Peprotech, Rocky Hill, NJ), 10 ng/ml human fibroblast growth factor 1 (R&D Systems, Minneapolis, MN), 6 µg/ml polybrene and incubated overnight with V5m3-400 vector particles at a multiplicity of infection of 40. The following day, 3 × 10^6^ cells were injected by tail-vein into SCD recipients (mixed 45.1/45.2; 1–4 months of age) that had received 400 cGy of total body irradiation. Selection of this dose was based on pilot studies showing that recipient SCD mice were acutely sensitive to higher levels of irradiation (600 cGy) with 50% mortality observed within the first week post-transplant. Occasionally, animals with 400 cGy received a failed injection of BM cells, but were maintained as controls. These animals survived for the duration of the study due to repopulation by endogenous BM indicating this dose was subablative. Furthermore, recipient SCD mice (*n* = 9) given 400 cGy but no rapamycin (Supplementary Figure S1) survived long-term but demonstrated <2% engraftment of donor cells indicating that survival was dependent on repopulation with endogenous BM cells. Transplanted mice were maintained under specific pathogen-free conditions with antibiotics (Baytril, 4 ml per 350 ml water, Bayer Healthcare LLC, Whippany, NJ) added to the drinking water for 2 weeks after transplant.

### Immunosuppression

The BERK SCD mice were characterized with a mouse strain-specific single nucleotide polymorphism panel (Mouse 384 SNP panel, Charles River Laboratories, Wilmington, MA). While donor and recipient strains shared the C57Bl/6 MHC locus (b haplotype), the strains differed at approximately 40% of other alleles, suggesting mismatch at minor histocompatibility loci. Therefore mice were not syngeneic, and thus, recipient animals were immunosuppressed the day before and for 2 weeks after BM transplantation by intraperitoneal (i.p.) injection of Rapamune (sirolimus; rapamycin; RAPA; Wyeth Pharmaceuticals, Philadelphia, PA) at a dose of 3 mg/kg per day.

### Hematologic and HbF protein analysis

Mice were sedated with isoflurane and bled from the orbital sinus using 75 µl microcapillary tubes (CDC Technologies, Oxford, CT). Procedures to determine hemoglobin levels and blood cell counts using an automated analyzer or measure expression of human γ-globin by cellulose acetate gel electrophoresis, high-performance liquid chromatography, and flow cytometry have been described.^58^

### Reticulocyte analysis

Reticulocyte counts were determined for 5–10 µl of blood stained with thiazole orange (Reticulocyte count reagent, Becton Dickinson, Franklin Lakes, NJ) and directly analyzed by flow cytometry for fluorescein isothiocyanate fluorescence or further processed to assay for intracellular HbF. For this, blood was treated for 10 minutes with 0.05% glutaraldehyde, washed and stained with allophycocyanin-conjugated antibodies to mouse CD71 (BD Biosciences, Franklin Lakes, NJ). Reticulocytes were identified as CD71 and thiazole orange double positive cells. Samples were then permeabilized in a solution of 0.1% Triton X100 and reacted with phycoerythrin-conjugated antibodies to human HbF and analyzed for PE fluorescence.

### Red blood cell half-life

Mice were injected via tail vein with 3 mg Sulfo-NHS-biotin (Life Technologies, Grand Island, NY) in 200 µl PBS. Blood was collected 4 hours after injection and 2, 4, or 6 days later, and 3 µl reacted with PE-conjugated streptavidin (Life Technologies) to quantify biotin-labeled RBCs by flow cytometry.

### Spleen colony-forming unit (CFU-S) assay

Lin- SCD cells (10–15 × 10^3^) transduced with V5m3-400 particles were injected by tail-vein into C57Bl/6 (Jackson Laboratories, Bar Harbor, ME) recipients conditioned with 950 cGy of total body irradiation. Two weeks after injection, mice were sacrificed and one to three discrete spleen colonies (CFU-S) dissected for each animal.

### DNA analysis for lentivirus gene transfer and copy number

Genomic DNA was prepared from spleen colonies or BM cells of transplanted mice sacrificed 24–28 weeks post-transplant using the Gentra Puregene DNA Extraction Kit (Qiagen, Valencia, CA). DNA (5 µg) was digested with *Eco*RI or *Bgl*II and Southern blot performed to identify presence of vector DNA or estimate VCN, respectively. A radiolabeled DNA probe was hybridized with the membrane and signal intensity of the band measured using a Molecular Dynamic Storm 860 Phosphoimager (Sunnyvale, CA) and its accompanying software.

### Urine osmolality

Transplanted SCD animals were housed of in the absence of food and water for 16–18 hours, urine was collected and osmolality was determined as described.^26^

### Organ pathology

At necropsy, mice were euthanized and spleen weights were recorded. Tissues collected for microscopic evaluation were fixed in 10% neutral buffered formalin, embedded in paraffin, sectioned at 4 μm and stained with hematoxylin and eosin (H&E) to identify architecture or Prussian blue to evaluate iron deposition.

### Statistical analysis

Continuous data were summarized as mean ± standard deviation unless specified. Associations between pairs of continuous variables were evaluated using Spearman’s rank order analysis. Comparisons between groups were made using the Exact Wilcoxon-Mann-Whitney test. Where indicated, Student’s two-tailed *t-*test was used to determine statistically significant differences between mean values and variances of different data sets. *P* values less than 0.05 were considered statistically significant. Data presentation and statistical analyses were conducted using SAS Version 9.4 (Cary, NC) and GraphPad Prism Software (GraphPad, San Diego, CA).

## Figures and Tables

**Table 1:** Gene transfer and expression of the V5m3-400 vector in sickle cell disease hematopoietic stem cells

*Parameters*	*n*	*Mean ± SD*	*Median*	*Min*	*Max*
Engrafted cells (%)^a^	15	34.7 ± 13.5	33	16	63
F-cells (%)^b^	21	68.5 ± 12.6	74	36	85
Vector copy per cell^c^	15	1.8 ± 0.9	2	0.4	3.2
HbF (%)^d^	21	33.2 ± 6.8	34	20	44

a Percentage of donor cells present in the peripheral blood that reacted with antibodies to CD45.1 or CD45.2 as determined by flow cytometry.

b Percentage of red blood cells that reacted with antibodies to HbF as determined by flow cytometry.

c Vector copy number for BM DNA as determined by Southern blot was multiplied by the percentage of donor cells detected in the peripheral blood to calculate vector copy per cell.

d Amount of HbF present in the peripheral blood as a percentage of total hemoglobin measured by high-performance liquid chromatography.

HbF, fetal hemoglobin.

**Table 2:** Therapeutic efficacy of the insulated γ-globin vector in sickle cell disease mice given subablative conditioning

*Transplant group*	*n*	*Hb (g/dl)*	*RBC (×10^6^/µl)*	*WBC (×10^3^/µl)*	*ANC (×10^9^/µl)*	*Urine mosm/kg*	*Spleen (g)*
Mock	18	7.7 ± 1.2	6.1 ± 0.7	23.7 ± 8.0	4.8 ± 2.4	1,529 ± 433	1.5 ± 0.5
V5m3-400	21	8.9 ± 0.9	7.5 ± 0.7	13.1 ± 4.2	3.0 ± 1.3	2,687 ± 479	0.9 ± 0.3
WT Bl6	14	13 ± 0.4	9.3 ± 0.4	7.4 ± 1.3	1.3 ± 0.3	3,370 ± 256^a^	0.1 ± 0.04^a^

Values represent the mean and standard deviation (SD). All data were obtained 4–5 months after transplantation.

ANC, absolute neutrophil count; Hb, total hemoglobin concentration; RBC, red blood cell; WBC, white blood cell.

a WT Bl6 urine concentrating ability and spleen size determined for (*n* = 5 mice).

**Table 3:** Association between the age of recipient mice at bone marrow transplantation and treatment outcomes

Analysis	HbF (%)	F-cells (%)	VCN/cell	Hb (g/dl)	RBC (×10^6^/µl)	WBC (×10^3^/µl)	ANC (×10^9^/µl)	Urine mosm/kg	Spleen (g)
Correlation	−0.03	−0.27	−0.56	−0.29	−0.20	+0.49	+0.62	−0.24	+0.43
*P* value	0.89	0.23	0.03	0.19	0.38	0.027	0.0035	0.30	0.048

Values calculated by Spearman’s rank order analysis.

ANC, absolute neutrophil count; Hb, total hemoglobin concentration; HbF, fetal hemoglobin; RBC, red blood cell; VCN, vector copy number; WBC, white blood cell.
