# Supplementary material for: Amelioration of murine sickle cell disease by nonablative conditioning and γ-globin gene-corrected bone marrow cells
Source: Mol Ther Methods Clin Dev. 2015 Dec 2;2:15045–. doi: 10.1038/mtm.2015.45 (PMC4667717; doi:10.1038/mtm.2015.45)
Supplement: Supplementary Figures [file mtm201545-s1.pdf]

Supplementary Materials for

Amelioration of Murine Sickle Cell Disease by Non-Ablative Conditioning and γ-globin Gene-Corrected Bone Marrow Cells

Tamara I. Pestina[1], Phillip W. Hargrove[1], Huifen Zhao[1], Paul E. Mead[2], Matthew P. Smeltzer[3], Mitchell J. Weiss[1], Andrew Wilber[4*], and Derek A. Persons[1*]

* To whom correspondence should be addressed. E-mail: awilber@siumed.edu

This Supplementary file includes Figures. S1-S5

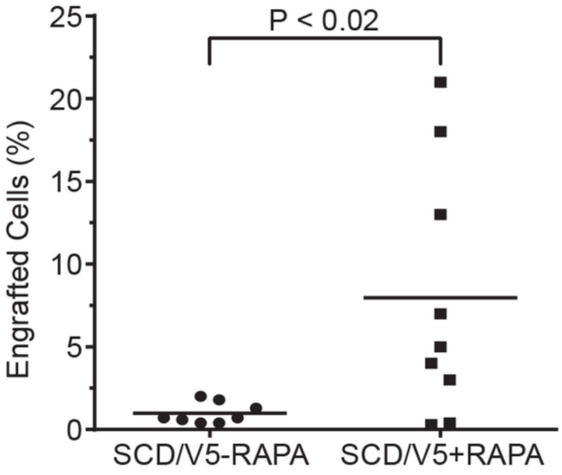

Figure S1. Donor-derived chimerism in SCD mice transplanted with gene corrected HSC is enhanced with rapamycin. Recipient SCD mice (n=9 mice per group) received either no immunosuppression (SCD/V5-RAPA) or intraperitoneal rapamycin (SCD/V5+RAPA) starting on the day before they were transplanted and for an additional two weeks. Symbols represent the donor chimerism reported as percentage engrafted cells in bone marrow of each recipient at 16 weeks after transplant. Mean values for each group are represented by the horizontal line and *P*-value calculated by two-tailed Student's *t*-test shown.

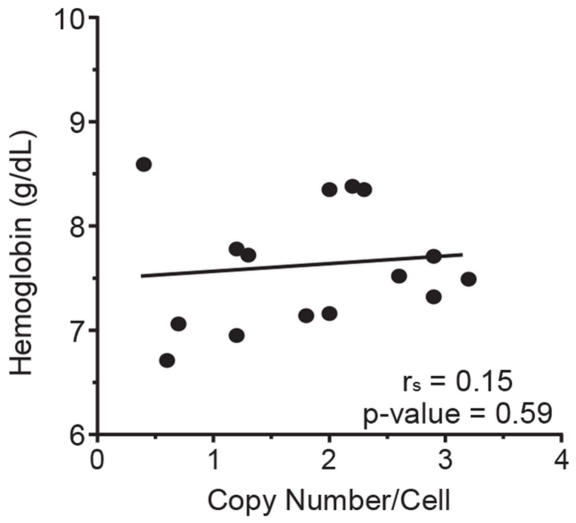

Figure S2. Relationship between vector copy number and levels of total Hb in peripheral blood of gene therapy treated SCD mice. Total hemoglobin levels at 18 weeks post-transplantation vs. vector copy number per cell. Regression lines (r_s) and p-values were calculated by Spearman's rank order analysis. Each symbol represents an individual mouse.

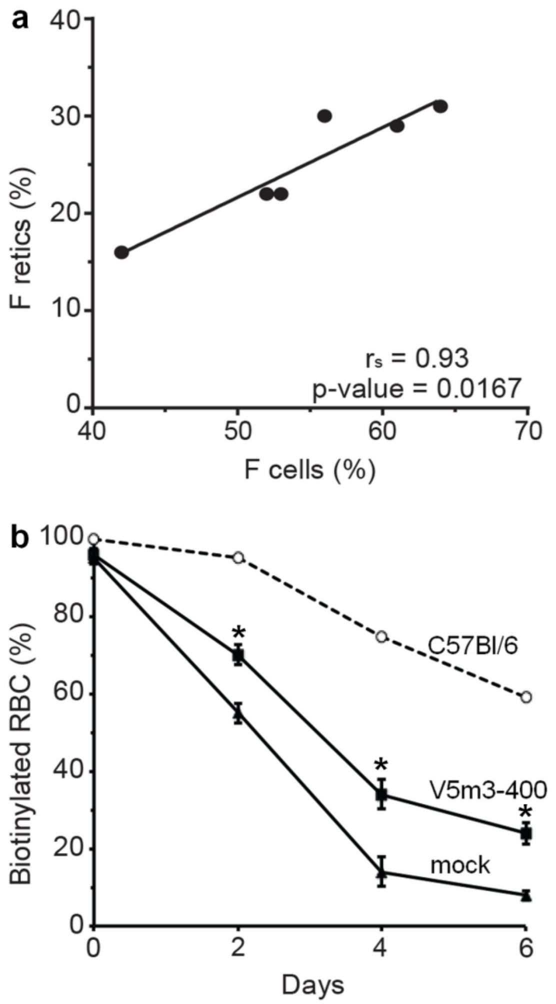

Figure S3. HbF expression in SCD mice improves RBC production and life-span. Sublethally irradiated BERK SCD mice were transplanted with mock (n=3) or V5m3-400-transduced (n=6) lin- BM cells as shown in Figure 1 of the main text. (**a**) Seven months after transplant, blood samples were collected from V5m3-400 treated mice and incubated with antibodies to CD71 and HbF or HbF alone to identify the proportion of reticulocytes and RBCs that expressed HbF. Each symbol represents an individual mouse with regression lines (r_s) and *P*-values calculated by Spearman's rank order analysis shown. (**b**) RBC survival determined by *in vivo* biotin labeling mock (n=3) versus V5m3-400 treated animals (n=6) and compared to wild type C57Bl/6 mice (n=2). Data were obtained 7 months post-transplantation and plotted as mean for wild type C57Bl/6 mice or mean $\pm$ sem for mock and V5m3-400 treatment groups. *p<0.01 mock vs. V5m3-400 by t-test.

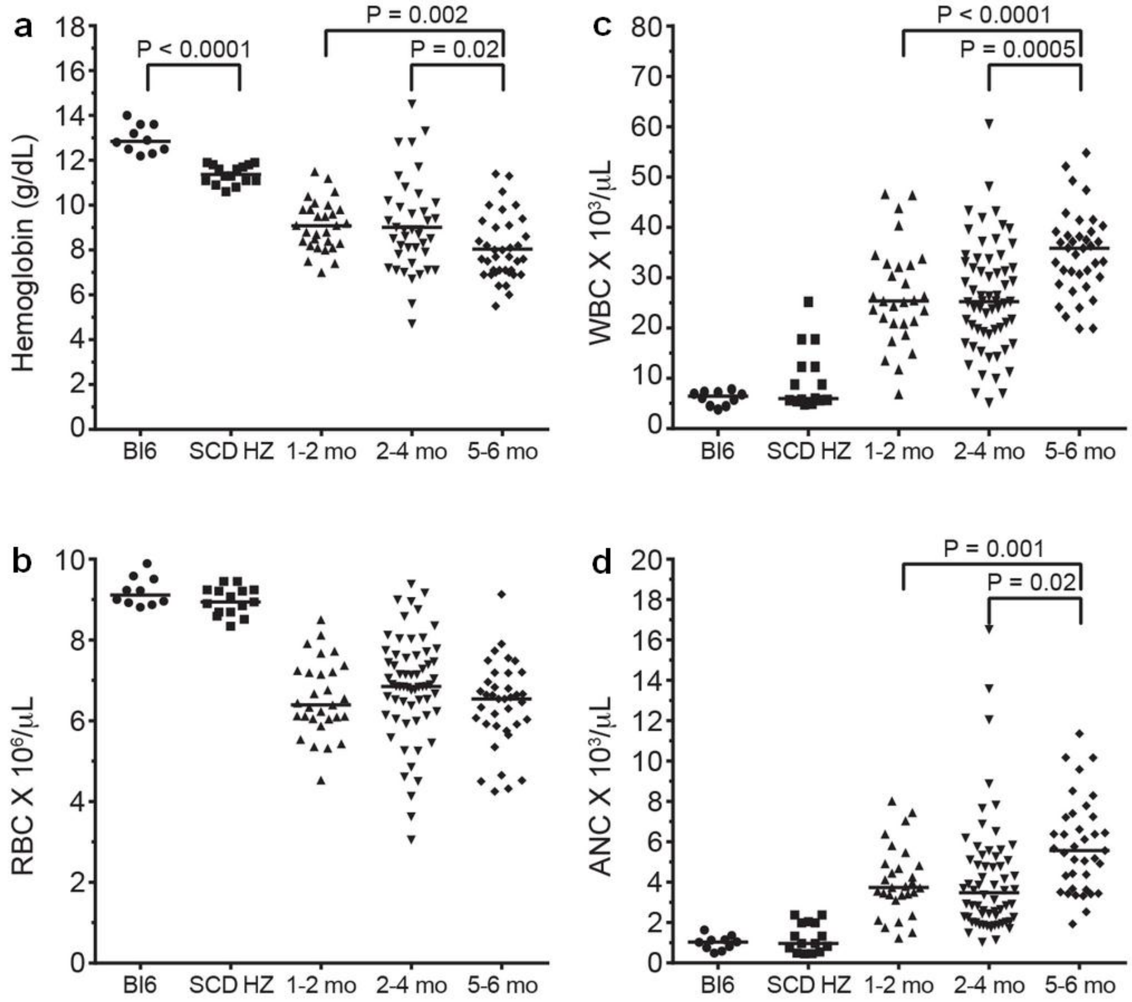

Figure S4. Anemia and hyperleukocytosis increase with age of untreated SCD mice. (**a**) Hemoglobin (Hb) levels, (**b**) red blood cell (RBC) counts, (**c**) white blood cell (WBC) counts, and (**d**) absolute neutrophil counts (ANC) for peripheral blood samples collected from normal C57/Bl6 mice (Bl6; n=10), SCD heterozygous littermates (SCD HZ; n=15) and SCD homozygous mice that were either 1-2 months old (SCD 1-2 mo; n=29), 3-4 months old (SCD 3-4 mo; n=62) or 5-6 months old (SCD 5-6 mo; n=37). Mean values for each group are represented by the horizontal line and *P*-values calculated by two-tailed Student's t-test are shown where differences between groups are significant.

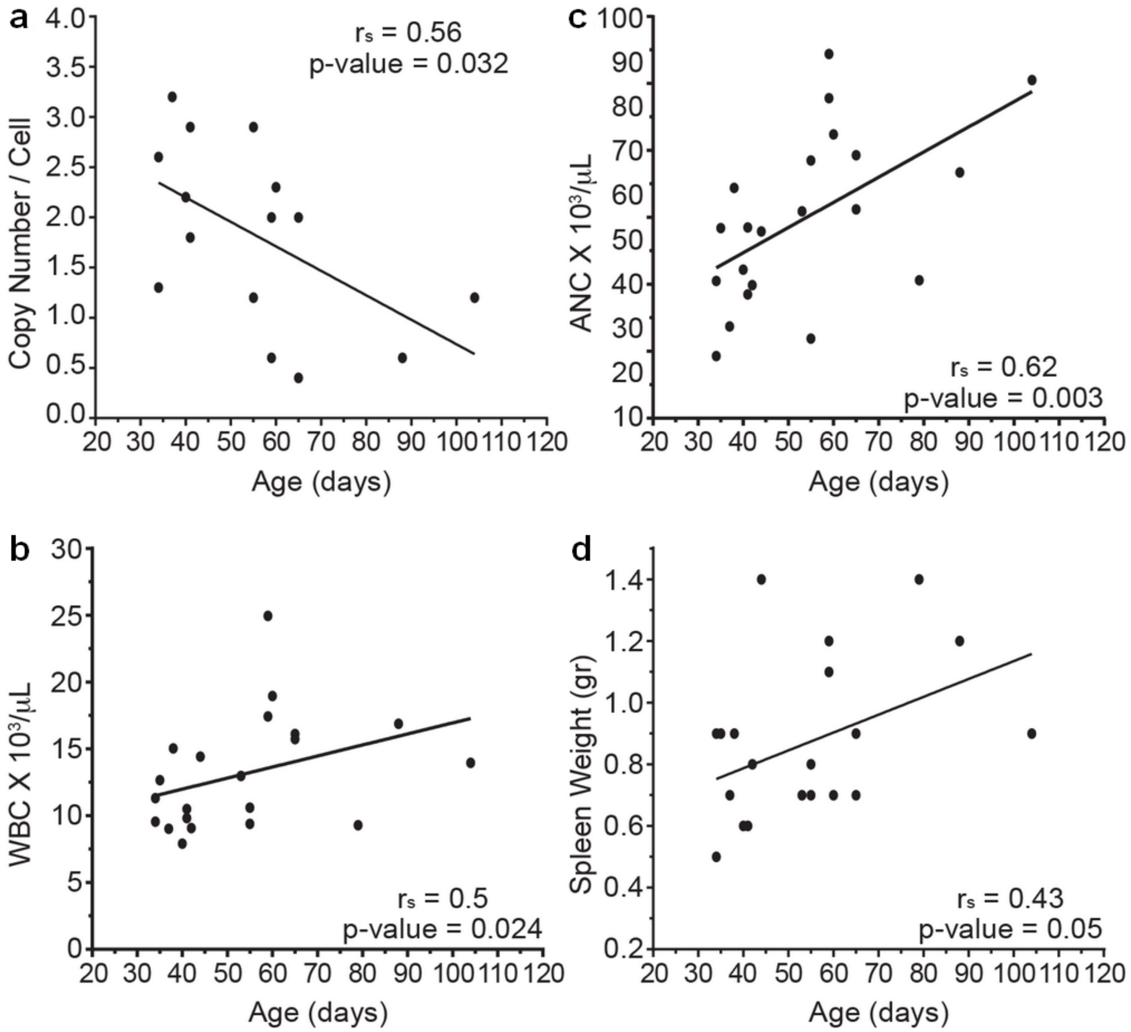

Figure S5. (Related to Table 3). Younger age SCD mice benefit from higher levels of gene transfer and less severe pre-existing inflammation. Vector copy number per cell (**a**), white blood cell (WBC) count (**b**), absolute neutrophil count (**c**) and spleen weight (**d**) were determined at 24-26 weeks after gene therapy performed according to Figure 1 of the main text and plotted against the age of recipient mouse at the time of time of bone marrow transplantation. Each symbol represents an individual mouse with regression lines (r_s) and *P*-values calculated by Spearman's rank order analysis shown.
